# Bariatric Surgery and Its Implications on Pharmacokinetics in Patients with Mental Illness: A Literature Review

**DOI:** 10.1192/j.eurpsy.2025.2061

**Published:** 2025-08-26

**Authors:** O. Alli-Balogun, K. Jafari, U. Anyeji, S. Gunturu

**Affiliations:** 1 Psychiatry, Bronxcare Health System, New York, United States

## Abstract

**Introduction:**

Obesity has reached epidemic proportions globally, significantly increasing the risk of cardiovascular diseases, diabetes, and various cancers. In the United States, as of 2017-2018, the prevalence of obesity among adults was approximately 42.4% (CDC, 2020). Worldwide, over 650 million adults were obese in 2016 (WHO, 2020).

Bariatric surgery, which includes a variety of procedures aimed at weight loss, While these surgeries primarily aim to induce weight loss and improve metabolic health, they also present challenges for patients taking psychotropic medications, such as antidepressants, antipsychotics, and mood stabilizers.

**Objectives:**

Understanding the Impact of Bariatric Surgery on Psychotropic Medication Management

1. Changes in Pharmacokinetics: Bariatric surgery can significantly alter the absorption, distribution, metabolism, and elimination (Smith et al., 2018).
2. Drug Interactions:Post-surgery changes in gastrointestinal physiology can impact drug interactions and the efficacy of psychotropic medications. (Alvarez et al., 2020).
3. The need for Monitoring and Dosing Adjustments:
4. Understanding Nutrient Deficiencies: can lead to deficiencies in essential nutrients like vitamin B12, vitamin D, iron, and calcium, which can exacerbate psychiatric symptoms or affect the metabolism of psychotropic medications (Courcoulas et al., 2020).

**Methods:**

A thorough literature review was carried out, with emphasis on research done within the the last 5 years.

**Results:**

On thorough reiew of literature, better outcomes resulted from implementing the following

1. Individualized Monitoring: Regular assessment of weight loss, nutritional status, and psychiatric symptoms can inform dosage adjustments to maintain therapeutic efficacy (Zachariah et al., 2015).
2. Consideration of Formulations: Immediate-release formulations may ensure consistent absorption and therapeutic effects due to altered gastrointestinal transit times (Lopez-Nava et al., 2017).
3. Collaborative Care:A multidisciplinary team, including bariatric surgeons, psychiatrists, pharmacists, and dietitians, can optimize medication regimens and address nutrient deficiencies (Smith et al., 2018).

Also considered the Benefits of Long-Acting Injectable (LAI) Psychotropics Post-Bariatric Surgery which improved the following

**Conclusions:**

Post Bariatric surgery,Clinicians must carefully monitor medication efficacy, adjust doses as needed, and manage nutrient deficiencies to ensure optimal mental health outcomes.

Ongoing research and multidisciplinary collaboration are essential to refining guidelines and improving the long-term management of these complex patient populations. By integrating personalized care strategies and considering LAI formulations, healthcare providers can optimize treatment outcomes for patients undergoing bariatric surgery.

**Disclosure of Interest:**

None Declared

